# Intrinsic Contribution of Perforin to NK-Cell Homeostasis during Mouse Cytomegalovirus Infection

**DOI:** 10.3389/fimmu.2016.00133

**Published:** 2016-04-06

**Authors:** Maja Arapović, Ilija Brizić, Branka Popović, Slaven Jurković, Stefan Jordan, Astrid Krmpotić, Jurica Arapović, Stipan Jonjić

**Affiliations:** ^1^Department of Histology and Embryology, Faculty of Medicine, University of Rijeka, Rijeka, Croatia; ^2^Center for Proteomics, Faculty of Medicine, University of Rijeka, Rijeka, Croatia; ^3^Department of Medical Physics, Clinical Hospital Rijeka, Rijeka, Croatia; ^4^Department of Oncological Sciences, Tisch Cancer Institute and the Immunology Institute, Icahn School of Medicine at Mount Sinai, New York, NY, USA; ^5^Faculty of Medicine, University of Mostar, Mostar, Bosnia and Herzegovina

**Keywords:** mouse cytomegalovirus, perforin, NK cells, bone-marrow chimera, Ly49H, m157, proliferation

## Abstract

In addition to their role as effector cells in virus control, natural killer (NK) cells have an immunoregulatory function in shaping the antiviral T-cell response. This function is further pronounced in perforin-deficient mice that show the enhanced NK-cell proliferation and cytokine secretion upon mouse cytomegalovirus (MCMV) infection. Here, we confirmed that stronger activation and maturation of NK cells in perforin-deficient mice correlates with higher MCMV load. To further characterize the immunoregulatory potential of perforin, we compared the response of NK cells that express or do not express perforin using bone-marrow chimeras. Our results demonstrated that the enhanced proliferation and maturation of NK cells in MCMV-infected bone-marrow chimeras is an intrinsic property of perforin-deficient NK cells. Thus, in addition to confirming that NK-cell proliferation is virus load dependent, our data extend this notion demonstrating that perforin plays an intrinsic role as a feedback mechanism in the regulation of NK-cell proliferation during viral infections.

## Introduction

Natural killer (NK) cells play a crucial role in the early stage of mouse cytomegalovirus (MCMV) infection; however, the contribution of NK cells varies among different mouse strains (1). In C57BL/6 mice, the activating NK-cell receptor Ly49H mediates resistance to MCMV infection, because of the specific binding of virally encoded m157 protein (2, 3). NK cells exert their function by releasing antiviral cytokines and by cytolytic mechanisms mediated by perforin and granzymes. Mice lacking the Ly49H receptor fail to exert significant virus control by NK cells during the early post-infection (p.i.) days, because of the fact that MCMV expresses immune evasion mechanisms able to avoid or decrease other means of NK-cell engagement (4–6). In addition, MCMV downregulates major histocompatibility complex class I (MHC-I) molecules to avoid detection by CD8^+^ T cells (7), but at the same time, the virally encoded *m04* protein escorts sufficient MHC-I complexes to the cell surface to ligate inhibitory Ly49 receptors and avoid NK-cell recognition (8). Thus, one can generalize that NK cells play a dominant role in MCMV control only in mice expressing Ly49H receptor and deletion of either the *m157* gene or blocking of the Ly49H receptor abolish the control in most of the organs (9–12). Perhaps the best evidence for the role of Ly49H/m157 interaction in MCMV control by NK cells is illustrated by strong selection pressure imposed by NK cells, resulting in numerous mutations and deletions in the *m157* gene after passing the virus through the Ly49H^+^ host (13).

Our research group along with other research groups has previously shown that NK-cell response to MCMV modulates subsequent CD8^+^ T-cell response and that both specific activation of NK cells and perforin-dependent mechanisms are involved (14–18). In C57BL/6 mice infected with wild-type (WT) MCMV, CD8^+^ T-cell response was markedly weaker compared to mice infected with the virus lacking *m157* gene, suggesting that specific virus control *via* Ly49H ligation dampens CD8^+^ T-cell response (19). It was previously shown that Ly49H ligation *via m157* enhances NK-cell proliferation (20). Perforin deficiency in NK cells compromises MCMV control, in spite of the fact that proliferation and production of cytokines were stronger than in WT NK cells expressing perforin (21). The immunoregulatory impact of NK cells on CD8^+^ T cells was still evident in perforin-deficient C57BL/6 (Prf1^−/−^) mice. Under these conditions, perforin-deficient NK cells regulate CD8^+^ T-cell response mostly by secreting inhibitory cytokine IL-10 (21). It remains unclear whether the enhanced proliferation of NK cells in Prf1^−/−^ mice is caused by a high virus load or if it represents a homeostatic function of perforin.

Here, we aimed to elucidate the immunoregulatory potential of perforin with the emphasis on NK-cell proliferation and differentiation during infection. For the same, we used a model of bone-marrow chimeras possessing NK cells with or without perforin and tested their response to MCMV. We found that in addition to virus load-dependent Ly49H^+^ NK-cell proliferation, perforin has an intrinsic role as a feedback mechanism in the regulation of NK-cell homeostasis during viral infections.

## Results

### Perforin Deficiency Enhances IFN-γ Secretion and Proliferation of NK Cells during Early MCMV Infection

**Figure 1:**
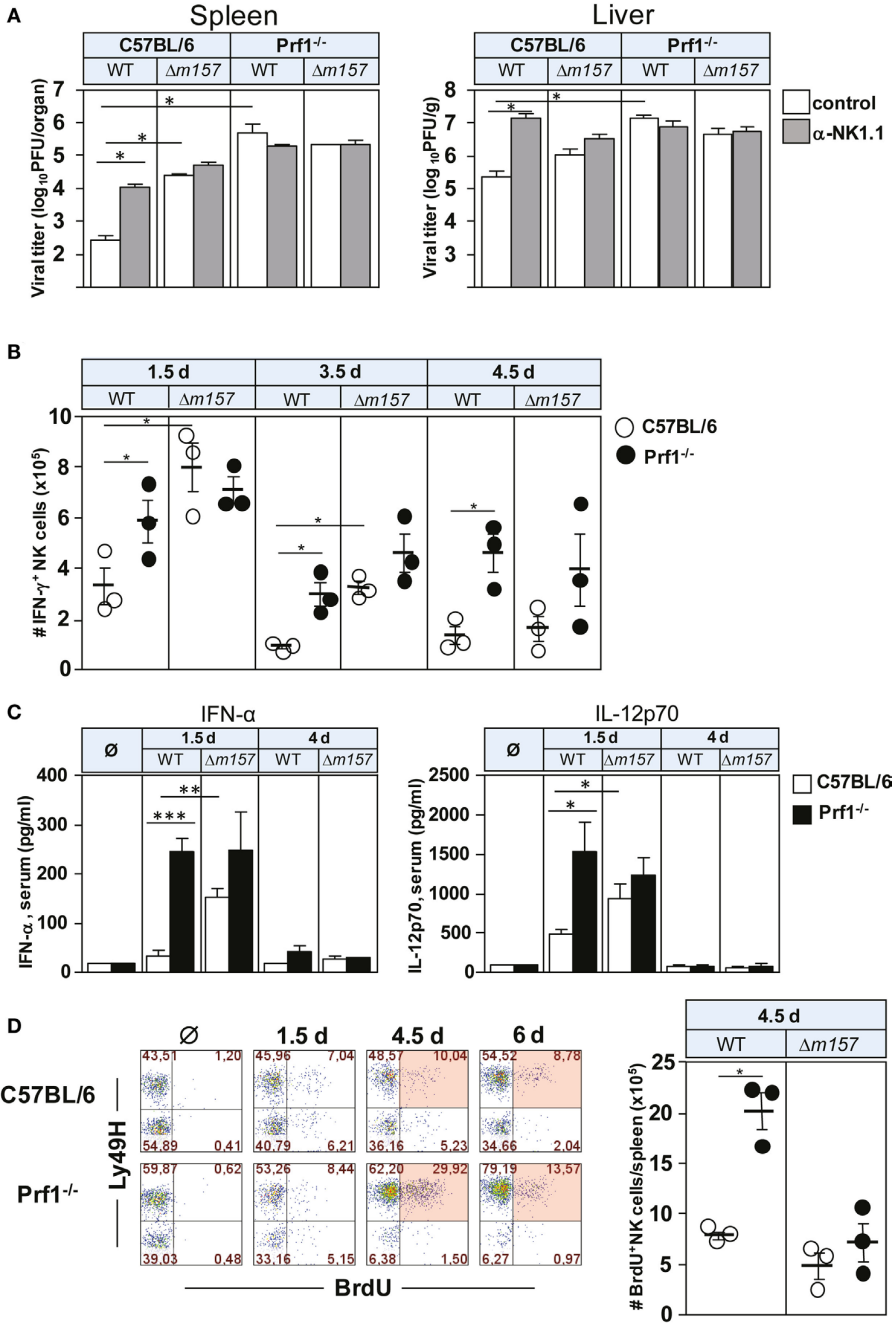
**Perforin deficiency enhances IFN-γ secretion and proliferation of NK cells during early MCMV infection**. C57BL/6 and Prf1^−/−^ mice were infected intravenously with 2 × 10^5^ PFU of indicated viruses. **(A)** Mice, either NK-cell depleted or NK-cell undepleted before infection, were euthanized 3 days p.i. and titers in spleen (per organ) and liver (per gram) were determined. **(B)** On days 1.5, 3.5, and 4.5 p.i., splenocytes were assessed for IFN-γ production by CD3ϵ^−^NK1.1^+^ NK cells. **(C)** On days 1.5 and 4 p.i., serum levels of the indicated cytokines were determined. **(D)** On days 1.5, 4.5, and 6 p.i., mice were i.p. injected with 2 mg of BrdU and euthanized 3 h later. The frequencies of BrdU^+^ CD3ϵ^−^NK1.1^+^ NK cells of both Ly49H^+^ and Ly49H^−^ subsets are depicted for wild-type (WT) MCMV infection (left). The number of BrdU^+^ CD3ϵ^−^NK1.1^+^ NK cells on day 4.5 p.i. following WT and Δ*m157* MCMV infection is shown (right). Representative data of at least two independent experiments with three to five mice per group are shown. Data are presented as means ± SEM. Asterisks denote significant values: *P < 0.05; **P < 0.01; ***P < 0.001.

To assess the impact of perforin on NK-cell response to MCMV, C57BL/6 and Prf1^−/−^ mice were infected with either WT MCMV or the virus mutant lacking *m157* (Δ*m157*), and virus titer was determined 3 days p.i. (Figure 1A). The proportion of mice in each group was subjected to depletion of NK cells by anti-NK1.1 monoclonal antibodies (mAbs). As shown previously (11, 22), MCMV control in C57BL/6 mice was almost completely dependent on Ly49H/m157 interaction. However, in perforin-deficient mice, the virus control by NK cells was essentially abolished, and no difference between WT virus and Δ*m157* mutant was found in spite of the fact that significantly more NK cells in perforin-deficient mice perforin produced IFN-γ, in comparison with WT control mice (Figure 1B). In C57BL/6 mice infected with Δ*m157* virus, we also found higher frequency of IFN-γ-producing NK cells in comparison with WT MCMV-infected mice, which correlates with a higher virus load and higher level of IFN-α and IL-12 in sera of Prf1^−/−^ mice (Figure 1C). However, in Prf1^−/−^ mice, higher level of cytokine production was observed irrespective of the virus used. Importantly, our results suggest that enhanced proliferation of NK cells in the absence of perforin is also driven by specific ligation of the NK-cell receptor Ly49H, because Ly49H^+^ cells proliferate much more strongly in mice infected with WT virus, as compared with virus lacking *m157* [Figure 1D; (20, 21)].

**Figure 2:**
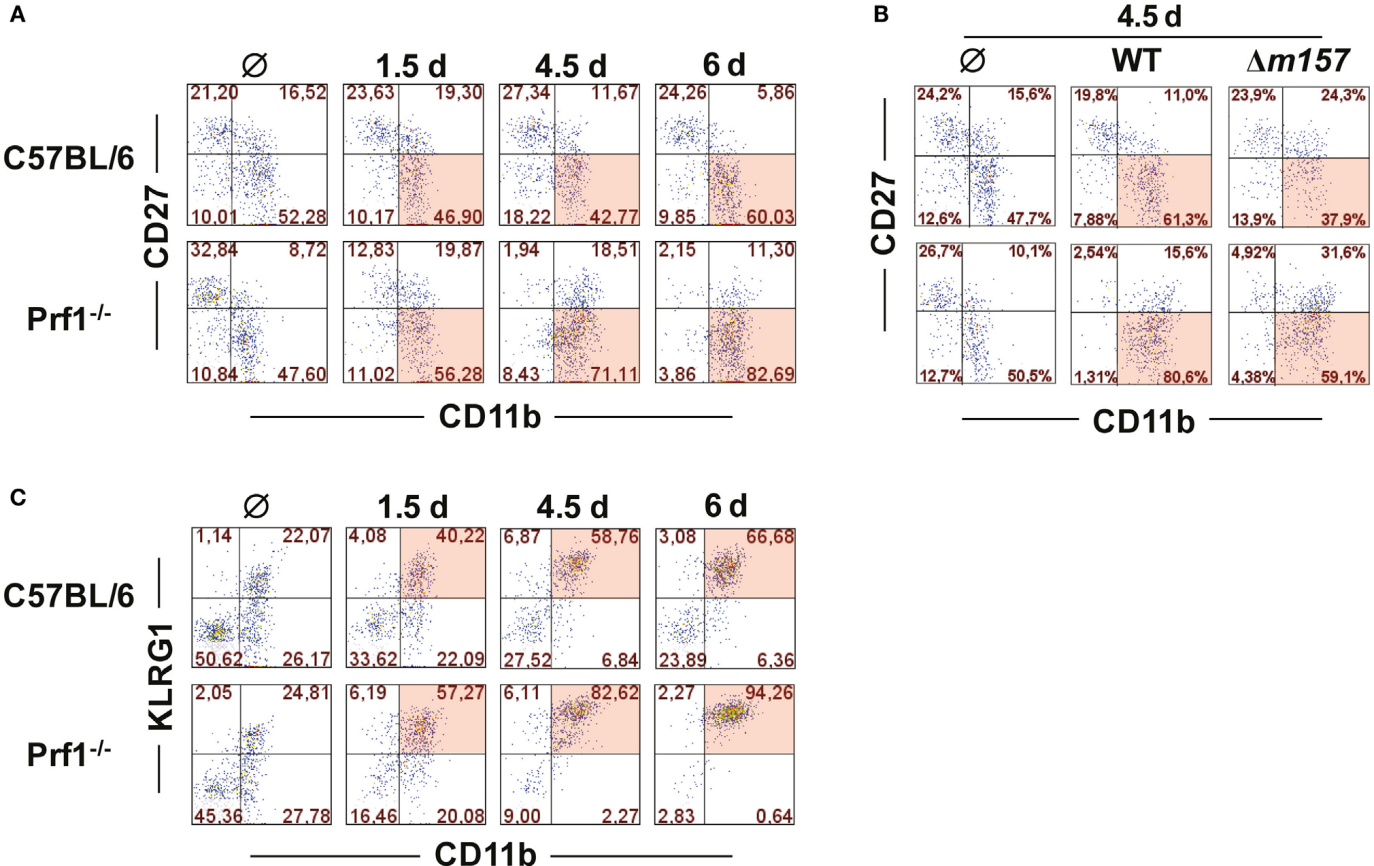
**Perforin deficiency enhances maturation of NK cells during early MCMV infection**. C57BL/6 and Prf1^−/−^ mice were infected intraperitoneally with 10^6^ PFU of MCMV **(A,C)** or intravenously with 2 × 10^5^ PFU of MCMV **(B)**. **(A)** At indicated time points, splenic CD3ϵ^−^NK1.1^+^ NK cells were analyzed for expression of CD27 and CD11b markers. **(B)** On day 4.5, maturation pattern of NK cells was determined for WT and Δ*m157* MCMV infection. **(C)** Frequencies of KLRG1 and CD11b coexpression on CD3ϵ^−^NK1.1^+^ NK cells at indicated time points p.i. are depicted. Representative data of at least two independent experiments with three to five mice per group are shown.

It is well established that MCMV infection drives maturation of NK cells toward a terminally differentiated phenotype (23). NK cells derived from MCMV-infected Prf1^−/−^ mice behaved in a similar fashion; however, the maturation was even more enhanced than in WT mice (Figure 2A). On day 6 p.i., the vast majority of NK cells in Prf1^−/−^ mice were of terminally differentiated CD27^−^CD11b^+^ phenotype. This pattern was observed even in mice infected with Δ*m157* virus, although our data indicate that Ly49H/m157 interaction further enhanced maturation of NK cells (Figure 2B). Further evidence for terminal differentiation of NK cells in the absence of perforin was provided by following the expression of KLRG1 at different time points after infection. As shown in Figure 2C, almost all NK cells in Prf1^−/−^ mice expressed KLRG1 on day 6 p.i., once again confirming acquisition of a completely mature phenotype (24).

In total, here, we showed that in the absence of perforin, NK cells proliferate far more and differentiate faster. In addition, a higher frequency of NK cells secrets IFN-γ, as compared to NK cells derived from control mice. It remains unclear whether these phenotypes of NK cells reflect the inability of Prf1^−/−^ mice to control the virus *via* NK cells, or if perforin plays an additional, so far uncharacterized, regulatory function.

### Enhanced Accumulation of Prf1^**−**/**−**^ NK Cells Is Their Intrinsic Function

**Figure 3:**
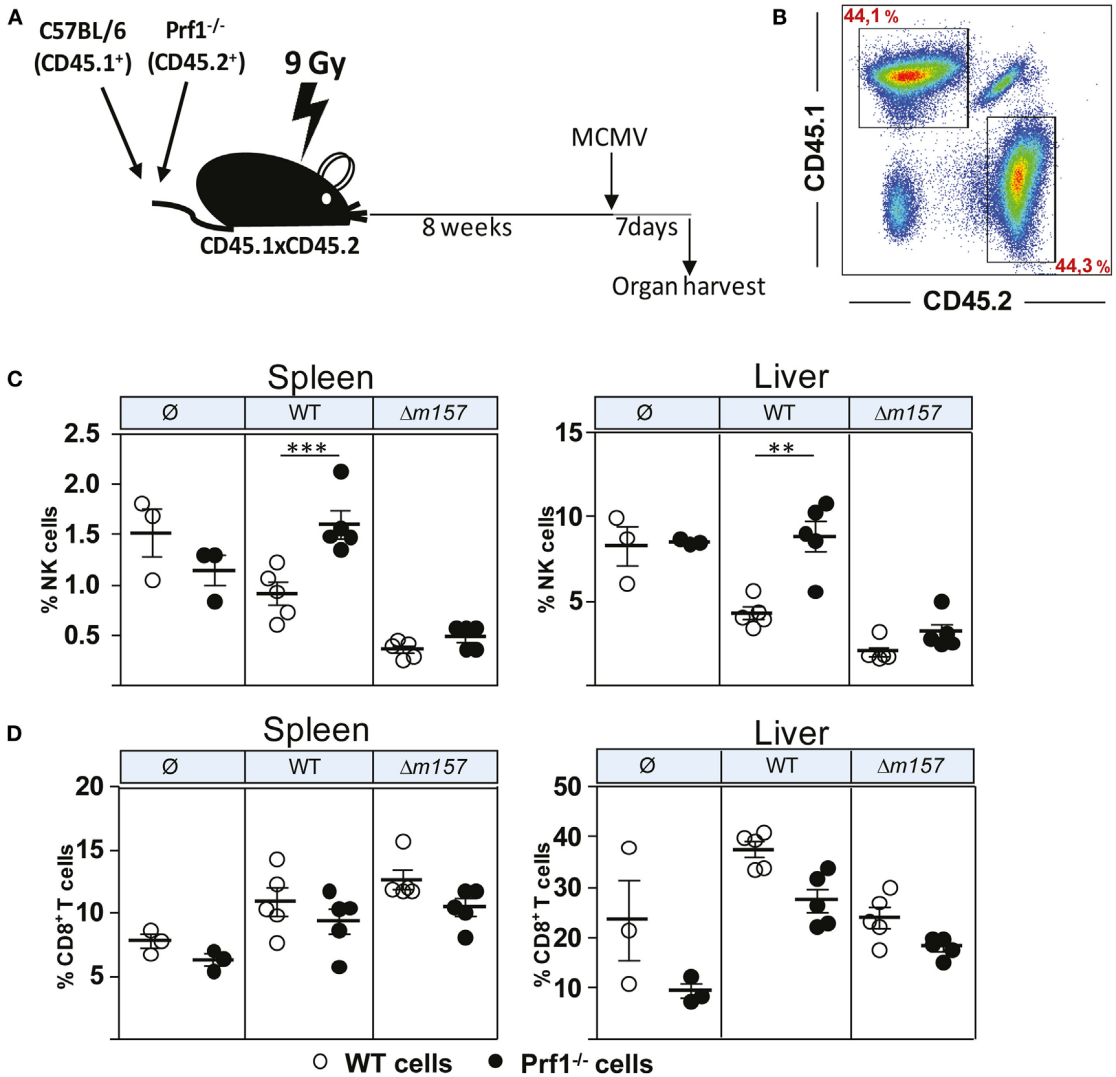
**Enhanced accumulation of perforin-deficient NK cells following MCMV infection in Prf1^−/−^/C57BL/6 chimeric mice**. **(A)** Mixed bone-marrow chimeric mice were prepared by transferring a 1:1 mixture of C57BL/6 (CD45.1^+^) and Prf1^−/−^ (CD45.2^+^) bone-marrow cells to γ-irradiated CD45.1 × CD45.2 mice. Chimeric mice were i.p. injected with 5 × 10^5^ PFU of WT or Δ*m157* MCMV and euthanized 7 days p.i. **(B)** Representative dot plot showing reconstitution efficiency of bone-marrow chimera. **(C)** Frequency of NK cells and **(D)** CD8^+^T cells in spleen (left) and liver (right) of Prf1^−/−^/C57BL/6 chimeric mice is shown. Representative data of two independent experiments with three to five mice per group are shown. Data are presented as means ± SEM. Asterisks denote significant values: *P < 0.05; **P < 0.01; ***P < 0.001.

Although the results described above clearly demonstrated the different functional properties of NK cells in the absence of perforin, we were not able to confirm that this is indeed the intrinsic effect of perforin on NK cells. This is because all these phenotypes (enhanced proliferation, faster maturation, and increased production of cytokines) could simply be a consequence of higher virus load in Prf1^−/−^ mice as compared to control C57BL/6 mice. To compare the proliferation and differentiation of Prf1^−/−^ and WT NK cells in the presence of identical virus loads, we generated bone-marrow chimeric mice (Figure 3A). Bone-marrow cells derived from C57BL/6 mice (CD45.1^+^) and Prf1^−/−^ mice (CD45.2^+^) were transferred at equal ratio into γ-irradiated C57BL/6 (CD45.1^+^CD45.2^+^) recipient mice. Eight weeks later, chimerism was confirmed (Figure 3B), and mice were infected with either WT, Δ*m157* MCMV, or left uninfected. Seven days later, mice were euthanized, and splenic and liver lymphocytes were analyzed (Figures 3C,D). In control uninfected chimeric mice, the frequency of Prf1^−/−^ NK cells was similar to WT NK cells, indicating no apparent impact of perforin on the generation of NK cells in steady-state conditions. However, in agreement with the results shown in Figure 1, a much higher frequency of Prf1^−/−^ NK cells was found both in spleen and liver in WT MCMV-infected chimeric mice, as compared to WT NK cells (Figure 3C). Although the absence of Ly49H/*m157* interaction resulted in a dramatic drop of NK-cell frequency, some differences between Prf1^−/−^ and WT NK cells were preserved. Lower frequency of splenic NK cells and decreased differences between perforin-deficient and perforin-sufficient NK cells in mice infected with Δ*m157* MCMV are likely a consequence of high virus load, as shown previously (19, 21). In addition, the results suggest that enhanced proliferation of perforin-deficient NK cells requires their stimulation *via* a specific receptor. Notably, no significant differences in frequency of Prf1^−/−^ and WT CD8^+^ T cells were found (Figure 3D). A slightly lower frequency of CD8^+^ T cells in Prf1^−/−^ mice was observed, regardless of infection. Thus, we demonstrated that, in chimeric mice, the enhanced accumulation and differentiation of Prf1^−/−^ NK cells is their intrinsic function, which becomes apparent during virus infection.

### Intrinsic Function of Perforin on NK-Cell Proliferation and Maturation Is Ly49H Dependent

**Figure 4:**
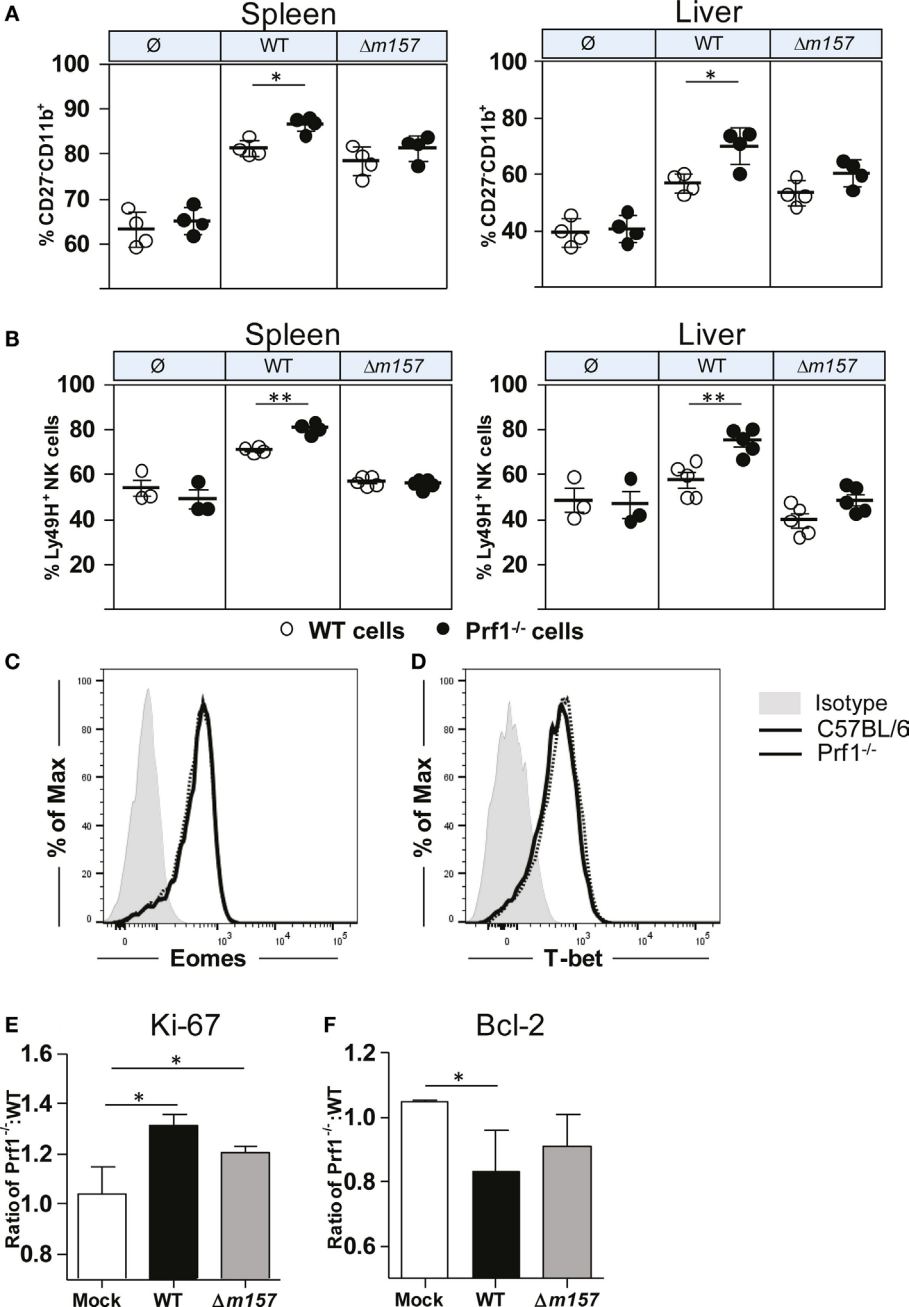
**NK-cell phenotype in Prf1^−/−^/C57BL/6 BM chimeric mice**. **(A)** Percentage of terminally differentiated CD27^−^CD11b^+^ and **(B)** Ly49H^+^ NK cells is shown in spleen (left) and liver (right) of WT MCMV, Δ*m157* MCMV, and mock-infected Prf1^−/−^/C57BL/6 BM chimeric mice. Representative histograms of **(C)** Eomes and **(D)** T-bet are shown. Relative expression of **(E)** Ki-67 and **(F)** Bcl-2 is shown calculated as ratio of expression of each marker in Prf1^−/−^ NK cells and C57BL/6 NK cells. Representative data of two independent experiments with three to five mice per group are shown. Data are presented as means ± SEM. Asterisks denote significant values: *P < 0.05; **P < 0.01; ***P < 0.001.

In Figure 1, we have shown that, during MCMV infection, NK cells of Prf1^−/−^ mice proliferated more strongly, allowing them to reach the terminally differentiated phenotype faster than NK cells from control C57BL/6 mice. To rule out the impact of a different virus load, next we tested maturation pattern of NK cells in bone-marrow chimeras (Figure 4A). Here again, we observed that the frequency of terminally differentiated perforin-deficient NK cells was higher than the frequency of terminally differentiated WT NK cells. Moreover, the results also showed that the Ly49H/m157 interaction does play a role, because the differences between Prf1^−/−^ and WT NK cells were not significant in mice infected with Δ*m157* virus. In line with this, the percentage of Ly49H^+^ Prf1^−/−^ NK cells was higher in comparison with Ly49H^+^ WT NK cells (Figure 4B). Thus, the intrinsic function of perforin on NK-cell proliferation and differentiation is more evident if NK-cell activation is driven through ligation of specific receptor.

What could be the mechanism by which perforin affects proliferation and maturation of NK cells? Higher frequency of Prf1^−/−^ NK cells in MCMV-infected mice could be either the consequence of changes in their transcriptome, enhanced proliferation (as indicated in Figure 1C), or prolonged survival. In bone-marrow chimeras, no differences in the expression of two key transcriptional factors regulating NK-cell development and differentiation, Eomes and T-bet, were detected between Prf1^−/−^ and WT NK cells (Figures 4C,D). Next, we tested the NK-cell expression of Ki-67, a marker of-cell proliferation, and Bcl-2, an anti-apoptotic protein. As shown in Figure 4E, Ki-67 was expressed in a greater proportion of Prf1^−/−^ NK cells than in WT NK cells. This was the case even in mice infected with the virus lacking *m157* gene. In contrast to Ki-67, expression of Bcl-2 appears to be lower in Prf1^−/−^ NK cells (Figure 4F).

**Figure 5:**
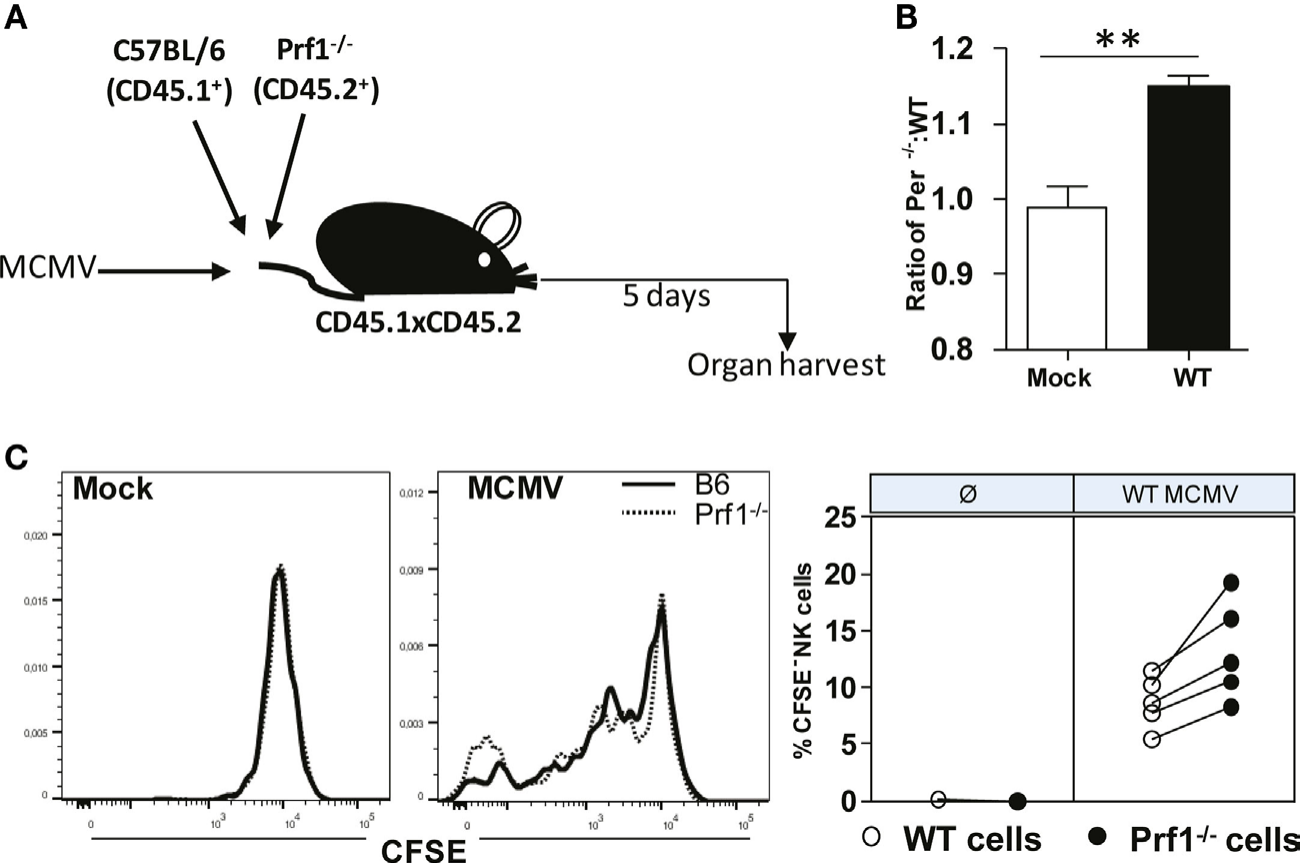
**Enhanced dilution of CFSE by perforin-deficient NK cells following MCMV infection in Prf1^−/−^/C57BL/6 chimeric mice**. **(A)** A 1:1 mixture of C57BL/6 (CD45.1^+^) and Prf1^−/−^ (CD45.2^+^) CSFE-labeled splenocytes were adoptively transferred to CD45.1 × CD45.2 mice that were infected with MCMV 3 h prior to transfer. **(B)** Ratio of number of adoptively transferred Prf1^−/−^ and C57BL/6 NK cells is depicted. **(C)** Proliferation of adoptively transferred Prf1^−/−^ and C57BL/6 NK cells in spleen was determined by analyzing CFSE dilution on day 5 p.i. Representative histograms for mock and MCMV-infected mice (left), and quantification of CFSE^−^ NK cells (right) are shown. Asterisks denote significant values: *P < 0.05; **P < 0.01; ***P < 0.001.

Our next goal was to confirm that Prf1^−/−^ NK cells indeed have a stronger capacity to proliferate compared to WT NK cells. To that aim, we used the adoptive transfer model in which we transferred CFSE-labeled Prf1^−/−^ (CD45.2^+^) and WT (CD45.1^+^) splenocytes into MCMV-infected CD45.1^+^CD45.2^+^ recipients (Figure 5A). Mice were euthanized on day 5 p.i., and CFSE dilution in NK cells was analyzed. In line with our data obtained in bone-marrow chimeras, we observed higher proportion of donor derived Prf1^−/−^ NK cells compared to Prf1^+/+^ NK cells following adoptive transfer (Figure 5B). In agreement with Ki-67 expression, CFSE was more diluted in Prf1^−/−^ NK cells than in Prf1^+/+^ NK cells (Figure 5C). Thus, our results confirmed that, in addition to serving as a major cytolytic molecule, perforin also plays an important intrinsic role by influencing proliferation capacity and differentiation of NK cells during virus infection.

## Discussion

Here, we describe a novel function of perforin in the regulation of NK-cell proliferation and differentiation during MCMV infection. In agreement with the previously described studies, we first confirmed that in the absence of perforin, NK cells proliferate more strongly upon infection, compared to NK cells from control mice expressing this cytolytic molecule. Furthermore, this enhanced proliferation is accompanied with increased IFN-γ secretion and NK-cell maturation. The enhanced proliferation is much more evident if activating NK-cell receptor Ly49H is engaged. Yet, it remains unclear whether this is only a consequence of higher virus load, because the virus control is almost completely compromised, and infection is accompanied by high level of proinflammatory cytokines in the absence of perforin. To provide equal conditions for both perforin-deficient and perforin-sufficient NK cells, we used bone-marrow chimeras and demonstrated that perforin-deficient NK cells still proliferate more and differentiate faster, confirming the intrinsic function of perforin in NK-cell response to viral infections. Note that these differences were absent in uninfected animals, confirming that perforin deficiency does not compromise NK-cell maturation and differentiation under normal conditions.

At first glance, our results are in disagreement with the results published by Lee et al., who showed that coculture of NK cells with cells expressing *m157* gene results in equal proliferation of perforin-deficient and perforin-sufficient NK cells (21). However, these two models are not fully compatible, because *in vivo* infection conditions provide a different microenvironment, which is apparently essential for the phenotype that we observed. Several mechanisms could explain higher levels of perforin-deficient NK cells: (i) higher virus load and stronger cytokine response, (ii) homeostatic elimination by cytolytic cells, and (iii) prolonged interaction and enhanced activation of NK cells *via* the intercellular synapses with the infected cells. Lee et al. showed that an increase in virus load correlates with enhanced proliferation of NK cells (21). However, in bone-marrow chimeras, perforin-deficient and perforin-sufficient NK cells are exposed to the same virus load and cytokine environment. Another way by which perforin deficiency could result in enhanced accumulation of NK cells could be as a consequence of decreased elimination of these cells by perforin-expressing effector cells. It is well established that perforin regulates immune cell homeostasis. During LCMV infection, perforin-mediated killing is involved in deletion of anergic antigen-specific CD8^+^ T cells (25). Furthermore, regulatory T cells can induce death of tumor infiltrating NK and CD8^+^ T cells in a perforin-dependent manner (26). NK cells can also eliminate other cells as a part of their homeostatic function. Crouse et al. have previously shown that, in mice lacking type I IFN receptor, CD8^+^ T cells are highly susceptible to NK-cell killing in a perforin-dependent manner (27). NK cells can also commit fratricide in certain conditions and by doing so participate in NK-cell homeostasis (28, 29). However, the differences in accumulation of NK cells between Prf1^−/−^ and WT mice should be abolished in chimeric mice. Although, in this work, we were unable to characterize the ultimate mechanisms by which perforin regulates NK-cell maturation, it is worth mentioning that the recent study by Jenkins et al. showing that perforin-deficient NK cells form prolonged synapses with target cells (30). This leads to repetitive calcium signaling and enhanced production of cytokines. Interestingly, perforin-deficient NK cells remained in synapse with their targets for a significantly longer period of time than granzyme A- and B-deficient NK cells, but there was no difference in the case of CD8^+^ T cells lacking these cytolytic molecules. In addition, as proposed by Sad et al., prolonged interaction because of inability of cytotoxic cells to kill target cells may be sufficient for sustained stimulation and activation of effector cells (31). It needs to be tested whether or not this could explain better survival and enhanced differentiation of perforin-deficient NK cells.

Negative feedback mechanisms are essential for any physiological function. Here, we described the novel regulatory function of perforin in homeostasis of NK cells during virus infection, and excluded several mechanisms that have been described to be involved in homeostatic regulation of NK cells and other effector cells. Further studies are required to fully elucidate this novel immunoregulatory role of perforin during viral infection.

## Materials and Methods

### Mice

C57BL/6, Prf1^−/−^ (32), C57BL/6 CD45.1^+^, and C57BL/6 CD45.1^+^CD45.2^+^ mice were housed and bred under specific-pathogen-free conditions at the Central Animal Facility of the Medical Faculty, University of Rijeka, in accordance with the guidelines contained in the International Guiding Principles for Biomedical Research Involving Animals. The Animal Welfare Committee at the University of Rijeka approved all animal experiments described in this paper. Eight- to 12-week-old mice were used in all experiments.

### Viruses

Mice were injected intravenously (i.v.) or i.p. with 2–5 × 10^5^ PFU of the tissue culture (TC)-grown virus in a volume of 500 μl of diluent (PBS or DMEM media). Bacterial artificial chromosome (BAC)-derived MCMV strain MW97.01 has previously been shown to be biologically equivalent to MCMV strain Smith (VR-1399) and is hereafter referred to as WT MCMV (33). In addition to MW97.01, the mutant virus lacking *m157* gene was used (9).

### Depletion of Lymphocyte Subsets and the Determination of Viral Titers *In Vivo*

The depletion of NK cells *in vivo* was performed by intraperitoneal injection of 300 μg of purified MAb PK136 (34) at 1 day before infection and 1 day p.i. For quantifying viral infectivity in organs, virus titers were determined by standard plaque assay, as described previously (35).

### Flow Cytometry and Intracellular Staining

Splenic leukocytes were prepared, as previously described, and in order to decrease non-specific staining, Fc receptors were blocked with 2.4G2 mAbs (36). The following *mAbs* were purchased from eBioscience or BD Pharmingen, and cell-surface staining was performed specifically for the following antigens: anti-CD3ϵ (145–2C11), anti-NK1.1 (PK136), anti-Ly49H (3D10), anti-CD27 (LG.7F9), anti-CD11b (M1/70), anti-CD8α (53–6.7), anti-IFN-γ (XMG1.2), anti-CD19 (1D3), anti-KLRG1 (2F1), anti-Ki-67 (SolA15), anti-Bcl-2 (10C4), and PE-Cyanine7-labeled streptavidin (SA-PE-Cy7). For the *in vivo* cell proliferation assay, mice were i.p. injected with 2 mg of bromodeoxyuridine (BrdU; Sigma) and euthanized 3 h later. To detect incorporated BrdU, splenic leukocytes were first stained for surface antigens and then fixed, permeabilized, refixed, treated with DNase I, and intracellularly stained according to the manufacturer’s protocol (BrdU flow kit; BD Pharmingen). For the detection of IFN-γ expression by NK cells, incubation was performed in RPMI medium supplemented with 10% of fetal calf serum (FCS; Gibco) for 5 h in the presence of 500 IU/ml of interleukin-2 (IL-2) at 37°C, with 1 μg/ml of brefeldin A (eBioscience) added for the last 4 h of incubation. After incubation, cells were first-surface stained and then fixed and permeabilized using Cytofix/Cytoperm solutions (BD Pharmingen) followed by intracellular IFN-γ staining, according to the manufacturer’s protocol. Staining of Ki-67 was done with the FoxP3 staining buffer set (eBioscience). For Bcl-2 intracellular staining, permeabilization and fixation of cells were done with the Intracellular Fixation and Permeabilization Buffer Set (eBioscience). Flow cytometry was performed on FACSCalibur and FASCAria (BD Bioscience; San Jose, CA, USA), and data were analyzed using the FlowJo software (Tree Star).

### Quantification of Serum Cytokine Levels

Serum levels of IFN-α were determined by an enzyme-linked immunosorbent assay (ELISA) kit for IFN-α (PBL Biomedical Laboratories), according to the manufacturer’s instructions. Serum levels of IL-12 were determined by Bio-Rad mouse cytokine multiplex assay, according to the manufacturer’s instructions (Bio-Rad Laboratories, Hercules, CA, USA).

### Bone-Marrow Chimeras

Bone-marrow chimeras were prepared using 8-week-old mice as donors and recipients. Briefly, C57BL/6 (CD45.1^+^CD45.2^+^) recipient mice were γ-irradiated with 9 Gy. After 24 h, recipient mice were injected i.v. with 10^7^ of donor bone-marrow cells containing a 1:1 mixture of C57BL/6 (CD45.1^+^) and Prf1^−/−^ (CD45.2^+^) bone-marrow cells. Recipient mice were maintained on antibiotic water containing enrofloxacin for 2 weeks following irradiation. Chimerism was evaluated 7 weeks post-transfer, and chimeras were used in experiments after 8 weeks of reconstitution.

### Adoptive Transfer of CFSE-Labeled Splenocytes

Splenic lymphocytes were isolated from C57BL/6 and Prf1^−/−^ mice and mixed at a ratio of 1:1. Splenocyte mixture was labeled with CFSE, as described previously (37). Briefly, cells were washed twice with PBS and suspended in 5-μM CFSE solution, followed by 12 min of incubation at 37°C. The cells were washed three times in complete RPMI media supplemented with 5% FCS and resuspended in complete RPMI. To verify the CFSE labeling of cells, samples were analyzed by flow cytometry. Recipient, non-irradiated C57BL/6 CD45.1^+^CD45.2^+^ mice were injected i.v. with 5 × 10^7^ of CFSE-labeled cells.

### Statistical Analysis

Statistical analysis was carried out using Prism 5 (GraphPad Software, La Jolla, CA, USA). Statistically significant differences between two data sets in cytokine assays and phenotype analyses were determined by the unpaired two-tailed Student’s *t*-test, and *P* values of <0.05 were considered significant. Differences in viral titers were determined by two-tailed Mann–Whitney *U*-test.

## Ethics Statement

The study was approved by the ethical committee of the Animal Welfare Committee at the University of Rijeka.

## Author Contributions

MA, IB, BP, AK, Stefan Jordan, and JA performed experiments. Slaven Jurkovic provided technical support. Stipan Jonjić, AK, MA, IB, BP, and JA designed experiments and wrote the manuscript.

## Conflict of Interest Statement

The authors declare that the research was conducted in the absence of any commercial or financial relationships that could be construed as a potential conflict of interest.
